# Retraction: Gonzalez, G.; Chen, L. EFA6 in Axon Regeneration, as a Microtubule Regulator and as a Guanine Nucleotide Exchange Factor. *Cells* 2021, *10*, 1325

**DOI:** 10.3390/cells11111721

**Published:** 2022-05-24

**Authors:** Gilberto Gonzalez, Lizhen Chen

**Affiliations:** Barshop Institute for Longevity and Aging Studies, Department of Cell Systems and Anatomy, UT Health San Antonio, San Antonio, TX 78229, USA; gonzalezg11@livemail.uthscsa.edu

The journal retracts the article “Retraction: Gonzalez, G.; Chen, L. EFA6 in Axon Regeneration, as a Microtubule Regulator and as a Guanine Nucleotide Exchange Factor” [1], cited above. Following publication, concerns were brought to the attention of the publisher regarding the improper reuse of text from previously published papers [2,3,4].

Adhering to our complaints procedure, an investigation was conducted that confirmed the overlap. This included Section 4, the first paragraph of Section 5.2, and Figure 3. The article is therefore retracted.

This retraction is approved by the Editor in Chief of the journal.

The authors agree to this retraction.
